# ^31^P magnetization transfer measurements of P_i_→ATP flux in exercising human muscle

**DOI:** 10.1152/japplphysiol.00871.2015

**Published:** 2016-01-07

**Authors:** Alison Sleigh, David B. Savage, Guy B. Williams, David Porter, T. Adrian Carpenter, Kevin M. Brindle, Graham J. Kemp

**Affiliations:** ^1^Wolfson Brain Imaging Centre, University of Cambridge School of Clinical Medicine, Cambridge Biomedical Campus, United Kingdom;; ^2^National Institute for Health Research/Wellcome Trust Clinical Research Facility at Cambridge University Hospitals NHS Foundation Trust, Cambridge Biomedical Campus, United Kingdom;; ^3^University of Cambridge Metabolic Research Laboratories, Wellcome Trust-Medical Research Council Institute of Metabolic Science, Cambridge Biomedical Campus, United Kingdom;; ^4^Fraunhofer MEVIS, Institute for Medical Image Computing, Bremen, Germany;; ^5^Department of Biochemistry, University of Cambridge, United Kingdom;; ^6^Cancer Research UK Cambridge Institute, Li Ka Shing Centre, University of Cambridge, Cambridge Biomedical Campus, United Kingdom;; ^7^Magnetic Resonance and Image Analysis Research Centre, University of Liverpool, United Kingdom; and; ^8^Department of Musculoskeletal Biology and MRC - Arthritis Research UK Centre for Integrated research into Musculoskeletal Ageing, Institute of Ageing and Chronic Disease, University of Liverpool, United Kingdom

**Keywords:** ^31^P magnetization transfer, saturation transfer, P_i_↔ATP exchange, exercising muscle

## Abstract

Fundamental criticisms have been made over the use of ^31^P magnetic resonance spectroscopy (MRS) magnetization transfer estimates of inorganic phosphate (P_i_)→ATP flux (V_Pi-ATP_) in human resting skeletal muscle for assessing mitochondrial function. Although the discrepancy in the magnitude of V_Pi-ATP_ is now acknowledged, little is known about its metabolic determinants. Here we use a novel protocol to measure V_Pi-ATP_ in human exercising muscle for the first time. Steady-state V_Pi-ATP_ was measured at rest and over a range of exercise intensities and compared with suprabasal oxidative ATP synthesis rates estimated from the initial rates of postexercise phosphocreatine resynthesis (V_ATP_). We define a surplus P_i_→ATP flux as the difference between V_Pi-ATP_ and V_ATP_. The coupled reactions catalyzed by the glycolytic enzymes GAPDH and phosphoglycerate kinase (PGK) have been shown to catalyze measurable exchange between ATP and P_i_ in some systems and have been suggested to be responsible for this surplus flux. Surplus V_Pi-ATP_ did not change between rest and exercise, even though the concentrations of P_i_ and ADP, which are substrates for GAPDH and PGK, respectively, increased as expected. However, involvement of these enzymes is suggested by correlations between absolute and surplus P_i_→ATP flux, both at rest and during exercise, and the intensity of the phosphomonoester peak in the ^31^P NMR spectrum. This peak includes contributions from sugar phosphates in the glycolytic pathway, and changes in its intensity may indicate changes in downstream glycolytic intermediates, including 3-phosphoglycerate, which has been shown to influence the exchange between ATP and P_i_ catalyzed by GAPDH and PGK.

magnetization transfer measurements using ^31^P magnetic resonance spectroscopy (MRS) of flux between inorganic phosphorus (P_i_) and ATP (P_i_→ATP), here called saturation transfer (ST), have been widely implemented for the putative assessment of mitochondrial function in skeletal muscle. In 2008, Kemp (13) drew attention to the striking fact that in resting muscle ST is an order of magnitude larger than the net rate of oxidative ATP synthesis that it was claimed to measure, a discrepancy too large to be compensated by the use of relative data presentations such as test/control or post/pre ratios. This and the separate point that a resting flux has no straightforward relationship to metabolic capacity (13, 16) have stimulated much recent debate over the interpretation of this measurement (2, 3, 11, 16, 27, 29, 35).

This discrepancy between ST and known or inferred rates of oxidative ATP synthesis (which we call here the “surplus” ST rate) in skeletal muscle is usually attributed to a rapid, near-equilibrium P_i_↔ATP exchange catalyzed by the glycolytic enzymes GAPDH (EC 1.2.1.12) and phosphoglycerate kinase (PGK; EC 2.7.2.3). Some early ST measurements of P_i_→ATP flux in *Saccharomyces cerevisiae* (5, 6, 9) provided evidence for a GAPDH/PGK-mediated exchange contribution. In addition, an in vitro study (8) showed that this GAPDH/PGK couple could, with simulated levels of enzymes and substrates, catalyze sufficient P_i_-ATP exchange to explain data obtained in glucose-perfused rat heart. This was subsequently confirmed by Kingsley-Hickman et al. (20) in intact perfused rat myocardium, when they manipulated glycolysis over a range of oxygen consumption rates. GAPDH and PGK activities are similar in human and rat heart and in rat skeletal and human intercostal muscle (31, 32). Although GAPDH activity is lower in human skeletal muscle compared with rat skeletal muscle (106 ± 28 vs. 294 ± 36 U/g tissue), it is still similar or greater than that of rat heart (31, 32).

Another potential explanation for a surplus P_i_→ATP flux relates to a mitochondrial P_i_-ATP exchange. LaNoue et al. (21) used ^33^P-radiolabeled tracers in isolated rat liver and heart mitochondria to demonstrate a significant ATP→P_i_ flux (i.e., in the reverse direction of ATP synthesis), and thus unidirectional P_i_→ATP rates in excess of the net ATP synthesis rate. In the transition from zero to maximal net ATP synthesis (in moving from state 4 to state 3 respiration) the P_i_→ATP flux doubled, whereas the reverse ATP→P_i_ flux decreased by >90%. Sheldon and Brindle et al. (30) also provided evidence for a mitochondrial P_i_-ATP exchange in vivo using ST measurements in yeast when they removed the glycolytic exchange catalyzed by GAPDH and PGK by lowering PGK expression using an attenuated promoter. Subtraction of the net glycolytic P_i_→ATP flux, estimated from measurements of glucose consumption, showed that overexpression of the adenosine nucleotide translocase (ANT) significantly increased the P_i_→ATP flux determined using ST measurements.

Other explanations proposed for the anomalously large P_i_-ATP flux, such as rapidly exchanging small pools of metabolites (2), remain speculative. For skeletal muscle the literature generally has been interpreted as favoring a glycolytic P_i_-ATP exchange mediated by the GAPDH/PGK couple (16, 27, 35), although it has been speculated (3, 11, 30) that in resting muscle with its low respiration rates, mitochondrial-associated P_i_-ATP exchange may become more prominent (21).

To investigate the determinants of this flux in vivo, we set out to define the effects of varying oxidative ATP synthesis rates on ST measurements in human skeletal muscle, which has been the main organ of interest in recent ST studies. There have been few studies of ST over a range of respiration rates: in stimulated rat hindlimb muscle (7), in lamb myocardium in vivo (26) and perfused rat myocardium (20), and in rat brain under varying levels of anesthesia (10). The rat hindlimb study (7) has been incorrectly cited (12, 22, 37) as supporting the validity of the resting ST as a measure of oxidative ATP synthesis. That study showed only that the P_i_→ATP flux in the stimulated muscle was not very different from the rates of net oxidative ATP synthesis observed in other studies that used similar experimental preparations and concluded that a glycolytic exchange contribution could not be ruled out, particularly in resting muscle. The lamb and rat myocardium studies (20, 26) found that the surplus P_i_→ATP flux remained approximately constant, or decreased, with increasing oxidative ATP synthesis rate. A retrospective comparison of the rat hindlimb results (7) with a range of published non-ST data resulted in similar findings (13). In the brain study (10), oxygen consumption was not measured. To study this relationship directly we designed a protocol to measure the steady-state rates of P_i_→ATP flux over a range of exercise intensities in human skeletal muscle and compared these with immediate postexercise rates of phosphocreatine (PCr) resynthesis, which are a measure of the suprabasal end-exercise mitochondrial oxidative ATP synthesis rate (15). We hypothesized that the surplus P_i_→ATP flux was a result of the exchange catalyzed by GAPDH and PGK and that this would remain unchanged, or decrease, with increasing oxidative ATP synthesis rate.

## METHODS

### 

#### Participants.

Each participant provided written informed consent and all studies were conducted in accordance with the Declaration of Helsinki. Ethical approval was granted by the UK National Research Ethics Service. Eleven healthy adult volunteers (7 men, 4 women) were recruited (age, 29.4 ± 2.8 yr; body mass index, 22.8 ± 0.9 kg/m^2^; means ± SE). Exclusion criteria included standard magnet contraindications, diabetes mellitus, cardiovascular disease, inability to understand protocol instructions, smoking, and taking medication or supplements known to affect energy metabolism.

#### Protocol.

Participants were recruited into *group A* or *group B*, except one volunteer who entered both groups. *Group A* consisted of nine volunteers who undertook a single ^31^P MRS scan with a workload predetermined using a fraction of their previously measured maximum voluntary contraction force. To test the feasibility of this exercise protocol in a variety of participants and over a range of ATP turnover rates, this fraction was varied among the volunteers. The three participants in *group B* undertook 4 ^31^P MRS scans on different days; the workload varied between visits, yielding sufficient PCr depletion (for measurement of PCr resynthesis) at low workloads while maintaining the exercise tolerability and minimal acidification at higher workloads.

On a prescanning visit, each volunteer's maximum voluntary contraction force was measured using a leg dynamometer (set to the same initial angle of exercise as in the magnetic resonance scanner), and all volunteers were shown an instruction video and given the opportunity to practice to ensure they were comfortable with the full in-scanner exercise protocol.

#### ^31^P MRS.

**Fig. 1.**
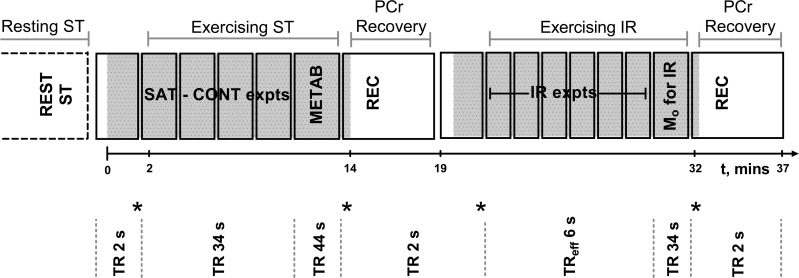
Schematic representation of the ^31^P magnetic resonance spectroscopy (MRS) exercise protocol. Solid lines symbolize sequence blocks, gray shaded regions correspond to when exercise occurred. Time from onset of exercise is illustrated by the timeline. In the first exercise section, once exercising steady-state conditions were met, spectra were obtained with saturation of the γ-ATP resonance and then control saturation placed equidistant to the inorganic phosphate (P_i_) resonance (SAT-CONT expts). Spectra were also obtained with a long repetition time (TR) of 44 s for calculation of metabolite concentrations (METAB). Following exercise cessation a phosphocreatine (PCr) recovery measurement (REC) was used to assess the immediate end-of-exercise oxidative ATP synthesis rate. Within the second exercise, once in steady state, the inversion recovery data were acquired with varying times between the inversion and subsequent excitation pulse (TIs) (IR expts) with an effective TR of 6 s, and a measure of M_o_ was also obtained. The four stars represent comparison sites for steady-state conditions.

Studies used a Siemens MAGNETOM 3T Verio (Erlangen, Germany) scanner, and each ^31^P MRS scan consisted of resting and exercising ST measurements, and assessment of postexercise PCr recovery kinetics (Fig. 1). The volunteers were positioned supine and a 6-cm-diameter surface coil (RAPID Biomedical, Rimpar, Germany) was attached at the right rectus femoris muscle (which was a location that gave the maximal PCr depletion/workload ratio). Precise coil relocation for participants in *group B* was obtained by using approximate anatomical distances and then accurately by three-dimensional fasciae landmarks. A magnetic resonance-compatible weight was attached to the right ankle (33) to provide the predetermined workload.

#### Resting ST measurement.

P_i_ magnetization was measured in the presence of selective saturation of the γ-ATP resonance (SAT) and compared with a control in which the irradiation frequency was placed symmetrical to the P_i_ peak (CONT) using a 1.32-ms BIR-4 adiabatic excitation (34) placed symmetrically between P_i_ and γ-ATP [repetition time (TR) = 24 s, receiver bandwidth (rBW) = 2,500 Hz, and number of acquisitions (NA) = 48 for each SAT/CONT]. The T_1_ of P_i_ with saturation of the γ-ATP resonance (T_1_′) was measured using an inversion recovery (IR) pulse sequence [7 TIs between 9 and 10,000 ms, effective TR (TR_eff_) = 6 s, NA = 12-20; TI was defined as the time between the inversion and subsequent excitation pulse, and TR_eff_ was defined as the time between the excitation and subsequent inversion pulse]. IR data were acquired in blocks that had the same TI, and the first spectrum of each group was eliminated. A fully relaxed spectrum was used to determine metabolite concentrations (NA = 12).

#### Exercising ST measurement.

Figure 1 outlines the exercising ST protocol. Knee extensions were performed (0.5 Hz) and spectra (TR = 2 s) acquired to ensure steady state was reached before ST acquisition. A soft target was attached to the apex of the scanner bore to prevent participant hyperextension, inhibit waning, and aid in maintaining a steady state. For ST measurements, triggered headphone instructions gave warning and then instructed participants to be still during the excitation and subsequent acquisition; any noncompliance resulted in exclusion of that spectrum. Two minutes of exercise preceded the first useable SAT spectrum and the participants were considered not to be in steady-state exercise if their average [PCr] at time = 80 s differed by >2 standard deviations of the end of exercise [PCr] (Fig. 1). To avoid significant acidification and to increase tolerability of the protocol, the exercise was split into two bouts (shaded regions in Fig. 1). Due to the potential for lengthening of the P_i_ T_1_ upon exercise (24), exercising ST parameters were similar to those of resting ST but with TR = 34 s, NA = 8 for each SAT/CONT; 5 TIs, NA = 6–15 for the T_1_ measurement, and a TR of 44 s and NA = 4 for the metabolite spectra.

#### Postexercise PCr recovery kinetics.

Ten spectra (TR 2 s) were obtained, which ensured that a steady-state magnetization had been reached (Fig. 1) before cessation of exercise and acquisition of spectra of the recovery kinetics (TR = 2 s, NA = 150). The PCr recovery rate constant, k_PCr_, was found using a two-parameter monoexponential fit, as described previously (25, 33). The suprabasal mitochondrial oxidative ATP synthesis rate was calculated from the immediate end-of-exercise rates of PCr resynthesis (V_ATP_), which were calculated as V_ATP_ = k_PCr_·[PCr_depleted_], where [PCr_depleted_] was determined as the difference between resting and exercising [PCr] from the fully relaxed metabolite spectra.

#### ^31^P MRS analysis.

**Fig. 2.**
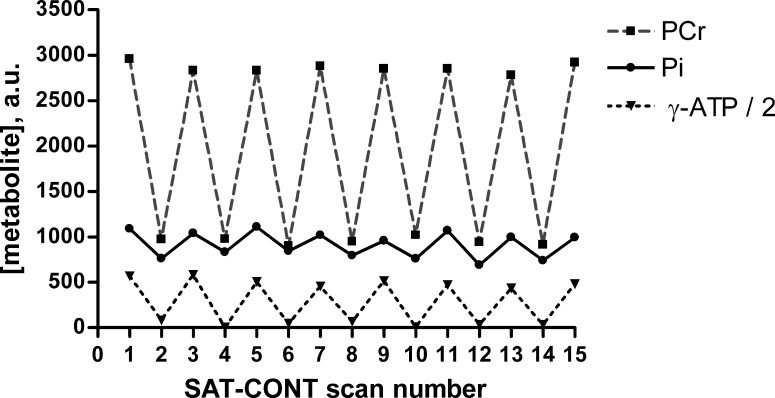
Individual time course of metabolite concentrations obtained during steady-state exercise with alternating γ-ATP and control irradiation. Representative (*group B* volunteer) metabolite concentration time course of PCr (squares), P_i_ (circles), and γ-ATP (triangles), obtained during steady-state exercise conditions with alternating frequency of saturation (SAT-CONT section in Fig. 1). Each *x*-axis point corresponds to a single spectrum. Even scan numbers correspond to spectra obtained with saturation of γ-ATP (SAT) and odd scan numbers to the equivalent control saturation frequency equidistant to P_i_ (CONT). Consecutive points are joined by gray dashed (PCr), solid black (P_i_), and dotted black (γ-ATP) lines to aid visualization.

All spectra were analyzed in jMRUI software (23) and phased and fitted to Lorentzian line shapes using the AMARES (36) algorithm with prior knowledge relating to resonant frequencies, j-coupling patterns, and relative amplitudes. Unlike the resting measurements, no averaging took place during exercise acquisitions and the SAT and CONT individual spectra were fitted (Fig. 2), thereby allowing for any change in the P_i_ chemical shift over time. IR spectra were averaged for each TI prior to fitting. The γ-ATP resonance from the corresponding resting or exercising metabolite spectra was used for calculation of metabolite concentrations, assuming an [ATP] of 8.2 mM (17). The intracellular pH was determined from the chemical shift of P_i_ relative to PCr (1), and the free concentration of ADP was calculated using established methods (1) assuming a total creatine pool of 42.5 mM (17). Due to the nonconventional line shapes of the phosphomonoester (PME) resonances, [PME] was determined by integration techniques. This involved using the averaged metabolite spectra and applying an optimized line broadening, equivalent to that of 0.75∗(line width of one singlet of the γ-ATP resonance doublet) before integrating the PME (5.9–7.5 parts per million) and γ-ATP resonances using the cut-and-weigh method. The Levenberg-Marquardt fitting algorithm within MATLAB (MathWorks, Natick, MA) was used to determine the T_1_′ of P_i_ from a two-parameter monoexponential fit, where M_o_ was fixed [from the SAT spectrum at rest and from M_o_-for-IR (Fig. 1) when exercising]. The first-order rate constant (k′) was determined according to the equation of Forsen and Hoffman: k′ = [(M_o_ − M_z_)/M_o_](1/T_1_′) and the P_i_→ATP flux (V_Pi-ATP_) by multiplication of k′ by the concentration of cytosolic P_i_. The exercising V_Pi-ATP_ component above the canonical net rate of oxidative ATP synthesis (surplus V_Pi-ATP_) was calculated as V_Pi-ATP_ − V_ATP_, where V_ATP_ was taken as the immediate end-of-exercise PCr resynthesis rate. Because V_ATP_ reflects the rate of suprabasal oxidative ATP synthesis, resting V_Pi-ATP_ was taken as the equivalent resting surplus V_Pi-ATP_ measure. The rate constant for postexercise PCr resynthesis, k_PCr_, was taken as a measure of muscle mitochondrial capacity (15).

#### Statistical analysis.

Statistical analysis was performed using IBM SPSS Statistics 21 software (IBM, Armonk, NY) with two-tailed significance set at *P* < 0.05. A paired-samples *t*-test was used to test for significant differences between resting and exercising conditions using one data pair per person. Spearman's correlation analysis was used to test for significant correlations because this required fewer assumptions that could be violated. Tests for significant correlations were performed using all data sets from *group A* and *group B* volunteers, and by averaging multiple scans from *group B* to give one data point per person. Quantitative data are expressed as means ± SE.

## RESULTS

All participants completed the exercise protocol and were fully compliant with the exercise instructions, resulting in no spectral exclusions. One scan (from a volunteer in *group B*) was lost due to broadband amplifier hardware failure and the ST data for another scan (of a volunteer in *group A*) were lost due to an incorrect saturation frequency. One participant in *group B* declined to provide a resting ST measure during the final visit but completed the exercise protocol, and another participant (in *group A*) failed to reach the steady-state exercise conditions, and that volunteer's ST exercise data were excluded. All remaining data were used.

Figure 2 illustrates the consistency of the steady-state exercise conditions and the changes in metabolite signals during saturation of the γ-ATP resonance in a representative individual.

**Fig. 3.**
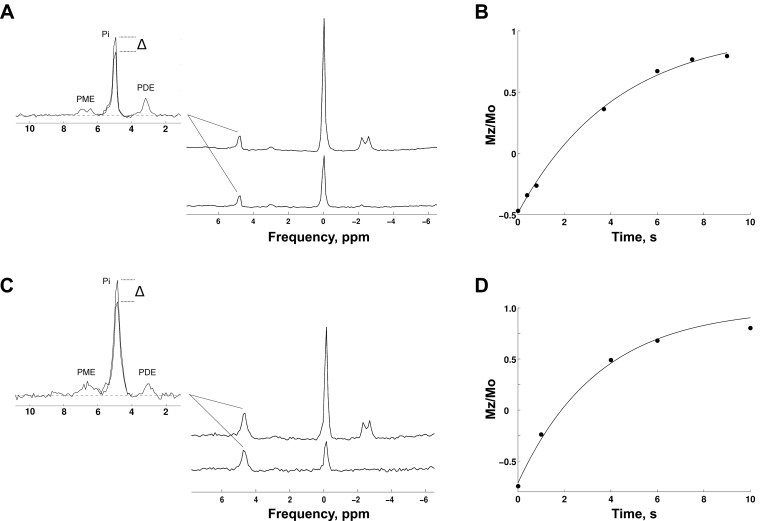
^31^P MRS measurements of P_i_→ATP flux at rest and during steady-state exercise. Representative saturation transfer (ST) spectra at rest (*A*) and during steady-state exercise (*C*), with saturation of the γ-ATP resonance (SAT) (*A* and *C*, *lower right*) and corresponding control spectrum (CONT) (*A* and *C*, *upper right*). The CONT spectra show the phosphomonoester (PME), P_i_, and phosphodiester (PDE) resonances (*A* and *C*, *left*), superimposed with the SAT P_i_ resonance to show the difference (Δ) in P_i_ resonance. Corresponding inversion recovery plot for measurement of the P_i_ T_1_ in the presence of γ-ATP saturation both at rest (*B*) and during steady-state exercise (*D*).

Figure 3 shows typical saturation transfer spectra and inversion recovery plots obtained at rest and during steady-state exercise.

Table 1 shows mean rest and exercise values of key ^31^P MRS measures. The overall mean fractional PCr depletion at steady-state exercise was 25 ± 3% (*n* = 20), and the mean postexercise PCr recovery rate constant (k_PCr_) was 1.86 ± 0.16 min^−1^ (*n* = 11, one k_PCr_ value per person). The mean change in pH_i_ at the end of exercise bout 1, compared with resting conditions, was −0.051 ± 0.016 (*n* = 20). Splitting of the P_i_ peak occurred only in one individual in *group A*, who also had the lowest exercise pH_i_ and the highest exercising V_Pi-ATP_ of 39 mM/min, and in this individual the two P_i_ resonances were fitted and then summed.

**Table 1.** Mean resting and exercising ST and PCr resynthesis measures

	Resting	Exercising	Paired-Samples Difference*	*P*†
ST, *n* = 9				
[P_i_], mM	3.37 ± 0.18	10.23 ± 1.10	6.9 ± 1.1	<0.001
T_1_′, s	4.5 ± 0.1	4.8 ± 0.3	0.3 ± 0.3	0.376
k′, min^−1^	2.98 ± 0.35	2.44 ± 0.16	−0.54 ± 0.39	0.204
V_Pi-ATP_, mM/min	9.8 ± 0.9	25.0 ± 2.9	15.1 ± 3.5	0.003
PCr resynthesis, *n* = 11				
[PCr], mM	32.9 ± 1.0	23.5 ± 1.2	−9.3 ± 1.2	<0.001
V_ATP_, mM/min	‡	16.5 ± 1.8		ND

ST, saturation transfer; P_i_, inorganic phosphate; T_1_′, apparent longitudinal relaxation time of P_i_ in the presence of saturation of the γ-ATP resonance; k′, first-order rate constant; V_Pi-ATP_, rate of P_i_→ATP flux; [PCr], concentration of phosphocreatine; V_ATP_, suprabasal oxidative rate of ATP synthesis determined from immediate end of exercise PCr resynthesis; ND, not determined.

Values are means ± SE. Data from volunteers in *group B* were averaged to provide one value per person to avoid inappropriate weighting.

* Paired-samples difference (exercising-resting).

† Paired-samples *t*-test to test for significant differences between rest and exercising conditions.

‡ For comparison with exercising, this is 0.0 because V_ATP_ reflects suprabasal oxidative ATP synthesis. The net rate of basal oxidative ATP turnover is thought to be approximately 0.5 mM/min (16).

Resting V_Pi-ATP_ did not correlate significantly with resting [P_i_], [ADP], or [H^+^] (all *P* > 0.2, *n* = 18, or *P* > 0.5, *n* = 10 averaging multiple scans from *group B*).

**Fig. 4.**
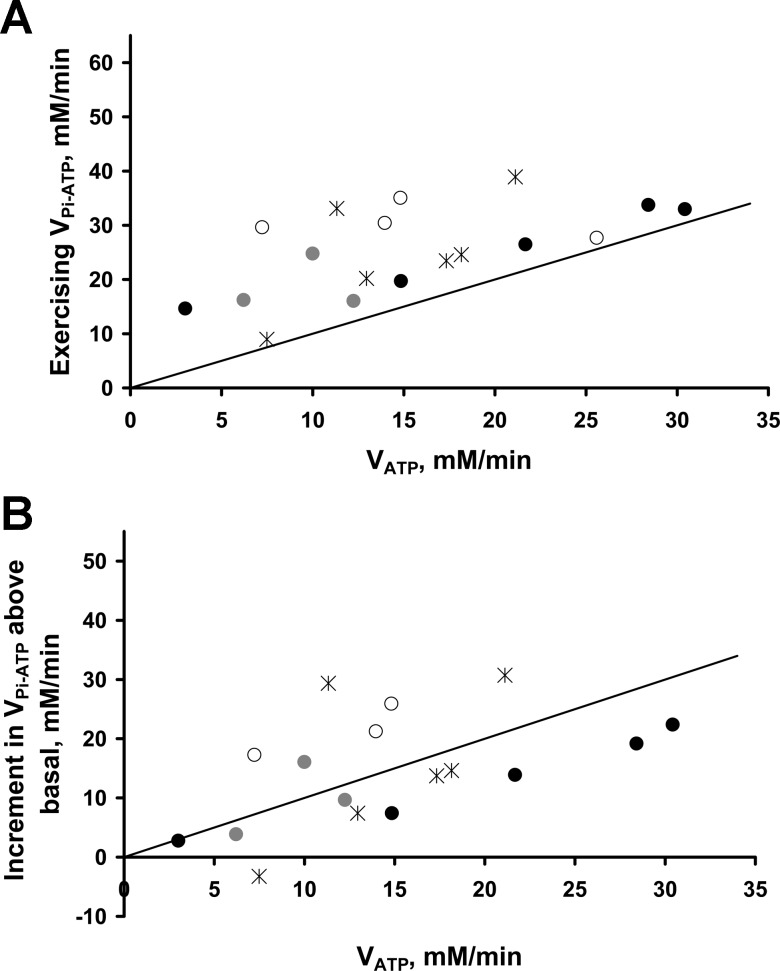
Steady-state rates of exercising P_i_→ATP flux and its increment above basal levels, compared with measures of oxidative ATP synthesis rates. Exercising steady-state rates of P_i_→ATP flux (V_Pi-ATP_) (*A*) and its increment above basal levels (*B*), plotted against oxidative ATP synthesis rates (V_ATP_) as measured from the immediate end-of-exercise PCr resynthesis rate. Black stars represent individuals in *group A*, and multiple scans of the three volunteers in *group B* are denoted by circles of black, gray, and white, respectively. The solid line represents unity equivalence of the two rates.

Figure 4 compares V_Pi-ATP_ during exercise with the immediate end-of-exercise rate of suprabasal oxidative ATP synthesis, measured as the initial PCr recovery rate (V_ATP_). Figure 4*A* shows the relationship between the two, with the line of identity for comparison; Figure 4*B* replaces the absolute V_Pi-ATP_ flux with the increment in V_Pi-ATP_ above the resting value. V_ATP_ correlated significantly with both the exercising V_Pi-ATP_ (*r* = 0.552, *P* = 0.017, *n* = 18) (Fig. 4*A*) and the suprabasal increment in V_Pi-ATP_ (*r* = 0.500, *P* = 0.041, *n* = 17) (Fig. 4*B*), but this fell outside statistical significance when averaging the multiple scans in *group B* (*r* = 0.65, *P* = 0.058 and *r* = 0.567, *P* = 0.112, respectively, *n* = 9). It is clear that surplus V_Pi-ATP_ (i.e., V_Pi-ATP_ − V_ATP_, the vertical distance above the line of identity in Fig. 4*A*), does not, on average, change over this range. Using all data points, exercising surplus V_Pi-ATP_ was not correlated with exercising [P_i_], [ADP], or [H^+^] (all *P* > 0.4, *n* = 18), but was correlated with exercising [P_i_] when averaging the multiple scans in *group B* (*P* = 0.02, *n* = 9).

**Fig. 5.**
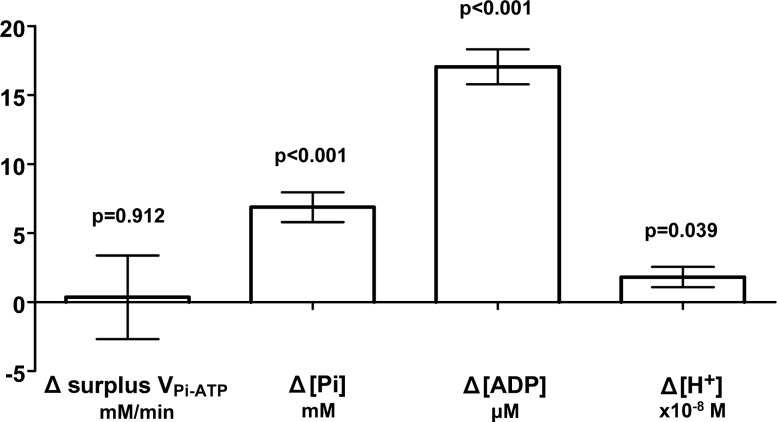
Paired-samples difference (Δ) in surplus P_i_→ATP flux and substrate concentrations of the enzymes GAPDH and phosphoglycerate kinase (PGK) between steady-state exercise and resting conditions. Paired-samples (*n* = 9) mean difference ± SE (exercising-resting values) for surplus V_Pi-ATP_ and substrate concentrations of the enzymes GAPDH and PGK; P_i_, ADP, and H^+^. Surplus V_Pi-ATP_ was calculated by subtracting the net rate of oxidative ATP synthesis, V_ATP_ (estimated as the immediate postexercise PCr resynthesis rate), from the rate of P_i_→ATP flux during exercise (V_Pi-ATP_) to provide an estimate of the component of the ST measurement not explained by suprabasal mitochondrial ATP synthesis. Data from volunteers in *group B* have been averaged to provide one value per person to avoid inappropriate weighting (hence *n* = 9). A paired-samples *t*-test was used to test for significant differences between resting and exercising conditions (*P* values shown).

Figure 5 shows this work rate-invariance for surplus V_Pi-ATP_ as the lack of a significant difference (*P* = 0.912, *n* = 9) between resting and exercising surplus V_Pi-ATP_, as assessed by a paired-samples *t*-test.

**Fig. 6.**
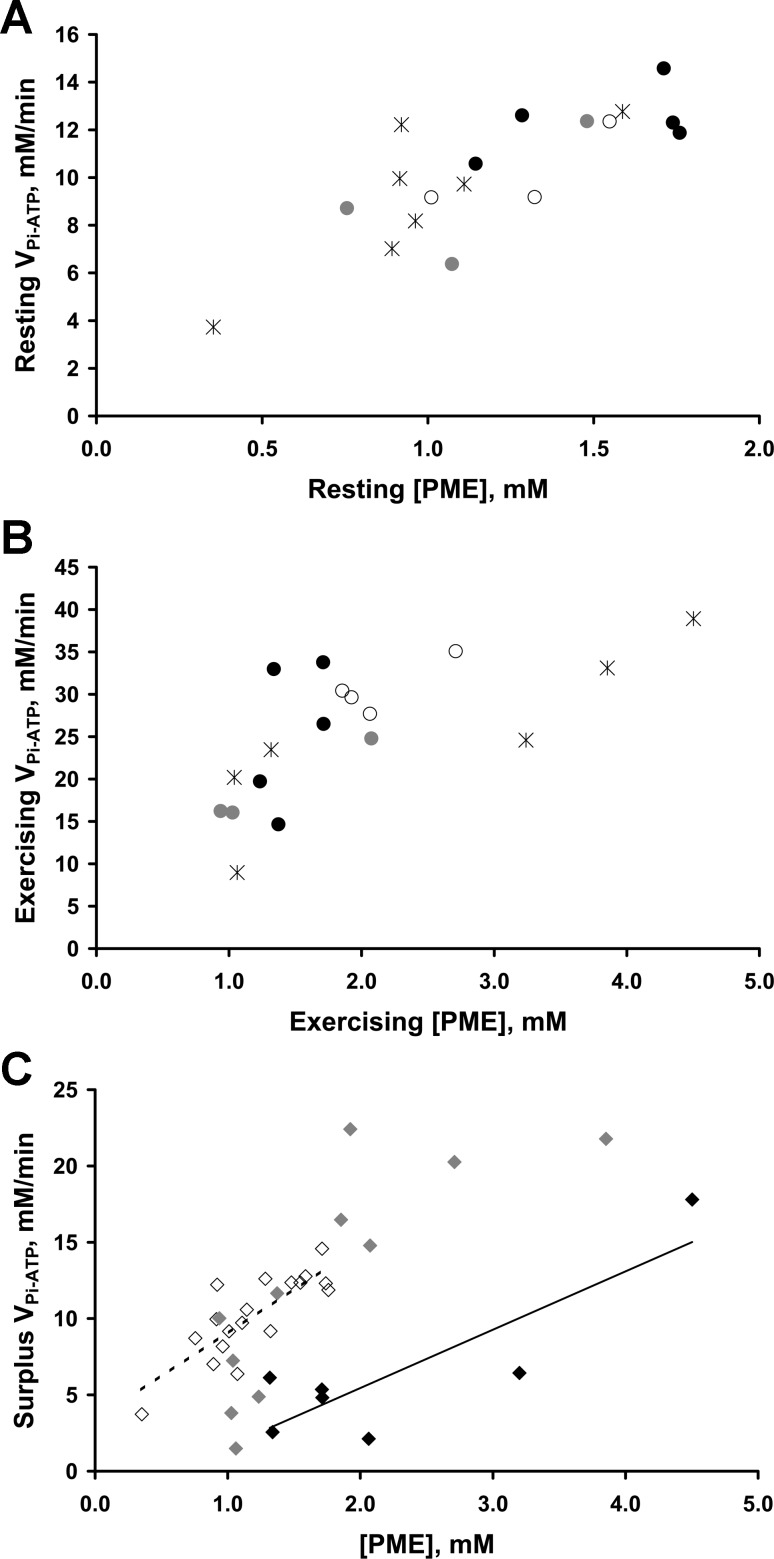
Relationship of the P_i_→ATP flux with the concentration of phosphomonoester (PME), at rest and during steady-state exercise. *A*: correlation of resting P_i_→ATP flux (V_Pi-ATP_) with resting [PME] (*r* = 0.740, *P* < 0.001, *n* = 18). *B*: relationship of exercising V_Pi-ATP_ with exercising [PME] (*r* = 0.730, *P* = 0.001, *n* = 18). As in Fig. 4, black stars represent the individuals in *group A*, and the multiple scans of the three volunteers in *group B* are denoted by circles of black, gray, and white, respectively. *C*: surplus V_Pi-ATP_ relative to [PME] at rest (white diamonds, *n* = 18) and during exercise (gray and black diamonds, *n* = 18). Surplus V_Pi-ATP_ was calculated by subtracting the rate of suprabasal oxidative ATP synthesis, V_ATP_ (estimated as the immediate postexercise PCr resynthesis rate), from the exercising V_Pi-ATP_. Resting V_Pi-ATP_ alone was used for the equivalent measure in resting muscle, where suprabasal ATP synthesis is by definition zero. Linear regression using both resting and exercising data (*n* = 36) found that in addition to [PME], V_ATP_ was also a significant negative predictor of surplus V_Pi-ATP_ (both [PME] and V_ATP_ *P* < 0.001). This is illustrated schematically here by dividing the exercising data into low (0.0–14.9 mM/min) and high (15.0–30.5 mM/min) exercising V_ATP_ groups denoted by gray and black diamonds, respectively. To aid visualization the dashed and solid black lines represent the trend lines for resting and high-exercising V_ATP_ groups, respectively, and highlight the association of V_ATP_ with reductions in surplus V_Pi-ATP_ for a given [PME].

Figure 6 examines the relationship between V_Pi-ATP_ and concentration of PMEs. First, resting V_Pi-ATP_ and resting [PME] were highly correlated (*r* = 0.740, *P* < 0.001, *n* = 18) (Fig. 6*A*), and also when averaging the multiple scans in *group B* (*r* = 0.770, *P* = 0.009, *n* = 10); these correlations remained significant after elimination of a possible outlier at low values. Second, exercising V_Pi-ATP_ and exercising [PME] were also highly correlated (*r* = 0.730, *P* = 0.001, *n* = 18) (Fig. 6*B*) (or *r* = 0.867, *P* = 0.002, *n* = 9 when averaging the multiple scans in *group B*). V_Pi-ATP_ comprises two components: a component due to net oxidative ATP synthesis (V_ATP_), and a surplus. This correlation of V_Pi-ATP_ during exercise with [PME] appears more closely related to the surplus exercising V_Pi-ATP_ (*r* = 0.534, *P* = 0.023, *n* = 18; or *r* = 0.65, *P* = 0.058, *n* = 9) (Fig. 6*C*, gray and black symbols) than the V_ATP_ (*r* = 0.261, *P* = 0.295, *n* = 18; or *r* = 0.067, *P* = 0.865, *n* = 9) contribution to exercising V_Pi-ATP_. Linear regression using both resting and exercising data (*n* = 36) found [PME] to be a significant predictor of surplus V_Pi-ATP_ (*R*^2^ = 0.291, *P* = 0.001). Supplementing [PME] with other measured variables revealed that V_ATP_, [P_i_], and [ADP] were also significant predictors of surplus V_Pi-ATP_, the models yielding the following results: [PME] and V_ATP_ (*R*^2^ = 0.572, [PME] and V_ATP_ both *P* < 0.001); [PME] and [P_i_] (*R*^2^ = 0.467, [PME] *P* < 0.001, [P_i_] *P* = 0.002); and [PME] and [ADP] (*R*^2^ = 0.369, [PME] *P* < 0.001, [ADP] *P* = 0.051). In these models V_ATP_, [P_i_], and [ADP] were all significant negative predictors of surplus V_Pi-ATP_ and hence had a significant effect in reducing the surplus V_Pi-ATP_. This can be seen in Figure 6*C*, where for a given [PME] the surplus V_Pi-ATP_ appears lower when exercising at high V_ATP_. The [PME] and V_ATP_ model yielded the highest correlation coefficient, and predicted a surplus V_Pi-ATP_ = 4.681 + 5.096 [PME] − 0.335 V_ATP_. When averaging the multiple scans in *group B* (*n* = 19), only [PME] alone (*R*^2^ = 0.675, [PME] *P* = 0.002) and [PME] with V_ATP_ were significant predictors (*R*^2^ = 0.787, [PME] *P* < 0.001 and V_ATP_, *P* = 0.02). Figure 6*C* also illustrates the apparent work-rate invariance of surplus V_Pi-ATP_ between resting and exercising conditions (as in Fig. 5), and suggests how this may be the result of the counteracting effects of increasing [PME] and decreasing V_ATP_ on the surplus V_Pi-ATP_. These relationships appear to underpin some of the variation in Figure 4.

Age correlated significantly with k_PCr_ (*r* = −0.679, *P* = 0.022, *n* = 11), but not with resting V_Pi-ATP_ (*P* = 0.44). Resting V_Pi-ATP_ did not correlate with k_PCr_ (*P* = 0.347). Also, k_PCr_ was not significantly correlated with exercising V_Pi-ATP_, its suprabasal increment, or the surplus exercise V_Pi-ATP_.

## DISCUSSION

The novel exercise protocol shown in Figure 1 has allowed ST measurements of steady-state P_i_→ATP flux over a range of workloads in human skeletal muscle with limited acidification. Minimizing acidification is important for two reasons. First, the relationships among pH, [PCr], and [ADP] imposed by the creatine kinase equilibrium mean that the interpretation of postexercise PCr recovery kinetics in terms of mitochondrial function is more straightforward. Specifically, a low pH is associated with slower PCr recovery for reasons that have nothing to do with any change in underlying mitochondrial function (15). Second, the use of a nonacidifying exercise protocol limits the contribution of net glycolytic ATP production to the measured P_i_→ATP flux. From the known stoichiometry of aerobic glycolysis (4), a reasonable approximation for the aerobic glycolytic rate in C6 units is 1/30 of the rate of ATP synthesis. This is an upper limit because it assumes, unrealistically (28), zero contribution by oxidizing fat. In our experiments the rate of oxidative ATP synthesis is estimated as V_ATP_, the initial postexercise rate of PCr resynthesis, and the highest measured value of V_ATP_ (Fig. 4) implies, therefore, an aerobic glycolytic rate of only ∼1 mM/min, or a net glycolytic ATP production rate of ∼2 mM/min. Pyruvate can also be reduced to lactate instead of being oxidized, and the rate of this can be estimated from the change in pH (14, 18). The average pH decrease was ∼0.05 units, which previous studies suggest would drive a H^+^ efflux rate of ∼0.4 mM/min (19). Consumption of H^+^ in the creatine kinase reaction can be ignored because there was no change in steady-state [PCr]. Therefore, the anaerobic glycolytic ATP production rate is ∼0.4 mM/min, representing a net glycolytic ATP production rate of no more than ∼2.4 mM/min.

The surplus P_i_→ATP flux remained approximately constant (Figs. 4 and 5) over the range from rest to the highest workloads undertaken (low to moderate respiration rates). This is consistent with inferences drawn from the stimulated rat hindlimb data (7, 11, 13). Similar invariance was also reported over low to moderate workloads in epinephrine-infused lamb myocardium; however, the fact that in that system P_i_ and ADP concentrations do not vary with workload (26) complicates meaningful comparison. In partial contrast, in glucose-perfused rat myocardium over moderate to high workloads the surplus P_i_→ATP flux appeared to decrease with increasing workload (20).

We also report for the first time the relationships between P_i_→ATP flux and PME concentration (Fig. 6), which included significant correlations of the [PME] with resting, exercising, and surplus P_i_→ATP flux. At rest and at the exercise intensities used in our study, the PME resonance is almost exclusively comprised of sugar phosphates (18), mainly glucose 6-phosphate (∼80%) fructose 6-phosphate (∼15%), and glucose 1-phosphate. The relationship of P_i_→ATP flux with a [PME] that contains major contributions from glycolytic pathway substrates appears to be consistent with a large glycolytic P_i_-ATP exchange contribution. GAPDH and PGK catalyze the coupled reaction: GAP + P_i_ + NAD^+^ + ADP ↔ NADH + H^+^ + 3PG +ATP.

While there was little change in [H^+^] there were substantial increases in [P_i_] and [ADP] between rest and exercise, yet the overall surplus P_i_→ATP flux remained unchanged (Fig. 5). One possible explanation for this is that the P_i_↔ATP exchange catalyzed by GAPDH and PGK may be also dependent on the concentration of the downstream glycolytic intermediate, 3-phosphoglycerate, [3PG], which would be expected to follow, at least to some extent, the concentration of the sugar phosphates represented by the PME resonance. Experiments with isolated GAPDH and PGK have shown a dependence of the exchange on 3PG concentration (8), although the effects of this are difficult to deconvolve from changes in the equilibrium concentrations of the other substrates of the GAPDH/PGK couple; nevertheless, linear regression of the data shows [3PG] to be a significant predictor (*P* < 0.001). Another factor relevant to the relationship between P_i_→ATP flux and [PME] might be the positive correlation of [PME] with [P_i_] found when considering all data points (at rest *P* = 0.038; exercising *P* = 0.012, *n* = 18); however, (notwithstanding its purely algebraic contribution; V_Pi-ATP_ = k′[P_i_]), resting [P_i_] was not significantly correlated with resting P_i_→ATP flux, nor exercising [P_i_] with exercising surplus P_i_→ATP flux (*n* = 18).

In this work we have defined the response of V_Pi-ATP_ in human skeletal muscle to large perturbations in the rate of ATP turnover, and partitioned it into the component due to net oxidative ATP synthesis, and what we have called surplus P_i_→ATP flux. The approach taken does not of course allow us to experimentally dissect contributions to the latter, although we have shown that net glycolysis cannot be a significant contribution. However, the correlations and surprising lack of correlations we have observed between fluxes and concentrations allow some mechanistic speculation. Taking the resting and exercising data together, [PME] was a significant positive predictor of surplus P_i_→ATP flux. Supplementing [PME], the suprabasal oxidative ATP synthesis rate was also found to be a significant predictor of the surplus flux, but acting in the opposite direction (Fig. 6*C*). The opposing effects of [PME] and V_ATP_ resolve into the overall invariance in surplus P_i_→ATP flux between resting and exercising conditions (Figs. 5 and 6*C*), and also explain some of the observed variation in P_i_→ATP flux (Figs. 4 and 6). Because little is known about [3PG] levels in skeletal muscle during exercise, we can only speculate that this may reflect a lower 3PG:PME ratio at higher net glycolytic flux.

Reflecting on all potential routes for transfer of magnetization between P_i_ and ATP, the P_i_ and γ-ATP resonances can exchange magnetization in the coupled reactions catalyzed by GAPDH and PGK, and possibly also via the ATP synthase (30), and via the unidirectional reactions of net ATP synthesis and breakdown. Net ATP synthesis, leading to direct transfer of magnetization between P_i_ and ATP, takes place in the reaction catalyzed by mitochondrial ATP synthase and, indirectly, following net glycolytic flux through the GAPDH and PGK reactions, although we have shown the latter to be insignificant under the conditions of this study. Glycolytic ATP synthesis in the reaction catalyzed by pyruvate kinase will not result in transfer of magnetization between P_i_ and γ-ATP. Net ATP breakdown, leading to direct transfer of magnetization between ATP and P_i_, will take place in muscle predominantly in the reaction catalyzed by the myofibrillar ATPase. All other routes for exchange of magnetization between P_i_ and the γ-phosphate resonance of ATP, most of which are less direct, are likely to be much slower.

In summary, we have demonstrated the feasibility of measuring P_i_→ATP flux in human exercising muscle over varying workloads. The surplus P_i_→ATP flux (that is, the amount by which it exceeds the known net mitochondrial ATP synthesis rate, estimated here from PCr recovery kinetics) is, on average, unchanged between rest and steady-state exercising conditions. This is in agreement with previous indirect inferences from rat skeletal muscle data, but seems surprising if (as commonly believed) the source of the surplus flux is P_i_-ATP exchange mediated by the glycolytic enzymes GAPDH and PGK, in view of the substantial changes in [P_i_] and [ADP] associated with increasing ATP turnover. However, some involvement of the GAPDH/PGK catalyzed exchange is suggested by the correlations observed between absolute and surplus P_i_→ATP flux and [PME] both at rest and during exercise. We speculate that this may be due to downstream changes in [3PG] concentration, which has been shown to influence GAPDH/PGK exchange kinetics in vitro.

## GRANTS

This work was funded by the Clinical Research Infrastructure Grant and the Siemens MAGNETOM 3T Verio scanner is funded by the NIHR via an award to the Cambridge NIHR/Wellcome Trust Clinical Research Facility. D.B. Savage is supported by the Wellcome Trust (091551).

## DISCLOSURES

No conflicts of interest, financial or otherwise, are declared by the authors.

## AUTHOR CONTRIBUTIONS

A.S. and G.J.K. conception and design of research; A.S. and D.B.S. performed experiments; A.S. analyzed data; A.S., K.M.B., and G.J.K. interpreted results of experiments; A.S. prepared figures; A.S. and G.J.K. drafted manuscript; A.S., D.P., K.M.B., and G.J.K. edited and revised manuscript; A.S., D.B.S., G.B.W., D.P., T.A.C., K.M.B., and G.J.K. approved final version of manuscript.
